# Estimating Health Condition Prevalence Among a Statewide Cohort with Recent Homelessness or Incarceration

**DOI:** 10.1007/s11606-025-09814-x

**Published:** 2025-09-02

**Authors:** Lucas Zellmer, Renee Van Siclen, Peter Bodurtha, Paul E. Drawz, Stephen C. Waring, Alanna M. Chamberlain, Behnam Sabayan, Steven G. Johnson, Karen Margolis, Rebecca Rossom, Katherine Diaz Vickery, Tyler N. A. Winkelman

**Affiliations:** 1 Health, Homelessness, and Criminal Justice Lab, Hennepin Healthcare Research Institute, Minneapolis, MN USA; 2 Hospital Medicine, Fairview Health Services, Minneapolis, MN USA; 3 Division of Nephrology and Hypertension, University of Minnesota, Minneapolis, MN USA; 4 Essentia Institute of Rural Health, Duluth, MN USA; 5 Department of Quantitative Health Sciences, Mayo Clinic, Rochester, MN USA; 6 Department of Neurology, Hennepin Healthcare Research Institute, Minneapolis, MN USA; 7 Division of Epidemiology and Community Health, School of Public Health, University of Minnesota, Minneapolis, MN USA; 8 Institute for Health Informatics, University of Minnesota, Minneapolis, MN USA; 9 HealthPartners Institute, Minneapolis, MN USA; 10 General Internal Medicine, Department of Medicine, Hennepin Healthcare, 701 Park Ave, P5.150, Minneapolis, MN 55415 USA

**Keywords:** homelessness, incarceration, electronic health record, chronic disease

## Abstract

**Background:**

Public health data systems have limited ability to provide timely, population-level information for people with severe and multiple disadvantages, such as individuals with recent homelessness or incarceration.

**Objective:**

To generate prevalence estimates for physical health, mental health, and substance use conditions in a statewide cohort that included individuals with recent incarceration or homelessness.

**Design:**

This observational cohort analysis was completed in July 2025 and used linked statewide electronic health record (EHR) and administrative data through the Minnesota Electronic Health Record Consortium (MNEHRC) and its Health Trends Across Communities project.

**Participants:**

Adults with an encounter at a MNEHRC-participating health system between 2021–2023.

**Main Measures:**

Statewide directly standardized, age and sex-adjusted, prevalence rates of 22 health conditions chosen by public health, healthcare, and research leaders in Minnesota, stratified by recent homelessness, jail incarceration, or prison incarceration.

**Key Results:**

This cohort included 4,362,645 individuals (53% female, 73% white), including 20,139 individuals with recent homelessness, 51,470 individuals with recent jail incarceration, and 4,889 individuals with recent prison incarceration. Individuals with recent homelessness or jail or prison incarceration had a higher prevalence of asthma (14.9%, 9.6%, 10.1% respectively, vs. 7.1%) and COPD (10.5%, 6.1%, 5.5%, respectively, vs. 3.0%) compared to the general population. Individuals with recent homelessness had the highest rates of mental health disorders compared to other included groups; recently homeless Black individuals had the highest recorded rates of psychotic disorder diagnoses (18.7%) across racial and ethnic groups experiencing homelessness. Substance use disorders among individuals with recent homelessness or jail or prison incarceration, including opioid use disorder (13.9%, 10.6%, and 13.6%, respectively, vs. 1.4%), were higher compared to the general population.

**Conclusions:**

Our findings highlight widespread disparities among people with recent homelessness or incarceration, particularly related to mental health and substance use conditions. Payment and delivery models that account for high levels of co-occurring health and social complexity are needed.

## INTRODUCTION

Monitoring the prevalence of health conditions in communities is critical for informing healthcare and public health policy and delivery.^1–3^ However, health condition prevalence data in the United States (US) are primarily based on survey methods, which often have a 2 to 3 year lag and lack the geographic granularity needed by local and regional healthcare systems and public health agencies.^4,5^ These limitations are particularly pronounced for populations with severe and multiple disadvantages, like individuals who have recently experienced homelessness or incarceration. People with recent homelessness or incarceration have a high burden of physical health,^6^ mental health,^7^ and substance use conditions^7,8^ compared with the general population, but they are typically absent from public health data sources.^9,10^ Robust data systems that include these populations are needed to inform policy and ultimately overcome substantial barriers to critically needed medical and behavioral health services, like vaccinations and medication for opioid use disorder.^11,12^

The Minnesota Electronic Health Record Consortium (MNEHRC) is a unique healthcare and public health collaboration that provides regularly updated health data on over 5.8 million Minnesotans.^13^ Through collaboration between healthcare systems, the Minnesota Department of Health, and other state and local agencies, the MNEHRC links electronic health record (EHR) data with statewide information on homelessness, incarceration, Medicaid enrollment, and mortality. Recently, our collaboration expanded previous work on COVID-19 to estimate and map health condition prevalence at the census tract level in Minnesota. Local public health agencies selected the initial health indicators and prioritized data that would be most likely to impact their agencies’ work over the next several years.

In this analysis using direct standardization, we provide prevalence estimates of physical health, mental health, and substance use conditions for a statewide population of people with recent homelessness or incarceration compared with the general population. To our knowledge, these are the first EHR-based statewide prevalence estimates among these populations in the US and could serve as a potential tool for future health policy and delivery changes.

## METHODS

### Cohort Design and Setting

MNEHRC includes 11 Minnesota healthcare systems that contribute data: Allina Health, CentraCare, Children’s Minnesota, Essentia Health, HealthPartners, Hennepin Healthcare, M Health Fairview, Mayo Clinic, Minneapolis VA, Sanford Health, and North Memorial Health. Each of the 11 healthcare care systems maintains their EHR data in the Observational Medical Outcomes Partnership (OMOP) common data model. Centralized R code is distributed to sites to query their database and generate descriptive data. We included any individual 18 and older with a Minnesota address in their health record and an encounter at a participating healthcare system (i.e., any outpatient, telehealth, emergency department, or inpatient visit) in the past three years (2021–2023, n = 4,362,645). Records were deduplicated so that patients seen at multiple health systems had their condition(s) counted only once.

This project was funded by the Minnesota Department of Health and was determined to be a public health surveillance project that did not require review from institutional review boards.

### Physical Health, Mental Health, and Substance Use Conditions

Records in the OMOP common data model use concept IDs that represent conditions in EHR data vocabularies like International Classification of Diseases (ICD) and Systematized Nomenclature of Medicine (SNOMED). We created code-sets of OMOP concept IDs that identify each condition. We first converted ICD-based diagnoses from the CMS Chronic Condition Warehouse^14^ to create a base set of OMOP concept IDs. We then used a combined metadata file from all sites to identify additional concept IDs related to the conditions of interest. After each codeset was created, a clinician reviewed codes for final inclusion or exclusion. Codes where there was uncertainty were adjudicated by a second clinician with expertise in OMOP and health services research. All codesets are available upon request. Hypertension includes a diagnosis and/or two outpatient blood pressure measurements ≥ 140/90. Diabetes mellitus includes a diagnosis and/or an A1c ≥ 6.5%. We describe substance-related conditions in this paper as substance use disorders because the diagnosis-based codesets generally represent problematic substance use and not use alone, though we did not have formal diagnostic assessment data available.

### Homelessness and Incarceration Data

Data on homelessness came from the Minnesota Homeless Management Information System which captures information regarding use of homeless services like street outreach, emergency shelter, and supportive housing.^15^ Federal housing programs (e.g. Section 8 housing) are not included. Data on incarceration came from records maintained by the Minnesota Department of Corrections on all admissions and discharges in Minnesota county jails and state prisons. Multiple healthcare systems that participate within the MNEHRC provide healthcare in county jails, while no healthcare system provides healthcare in state prisons. Diagnoses that are made during a jail encounter with a participating MNEHRC site are included in the analysis. For this analysis, we used a one-year lookback period for both homelessness and incarceration indexed to the date of the most recent analysis (HTAC version 2025.3, April/May 2025). For 2023, we report on 51,470 individuals of 82,040 who had any jail incarceration event. We report on 4,889 individuals of 11,936 who had a prison incarceration event in 2023. The denominators for jail and prison incarceration is inclusive of people currently incarcerated who would not have had community health care use. We report on 20,139 individuals of 50,184 who had experienced homelessness in 2023. The denominator for recent homelessness is inclusive of children who were not reported in this analysis.

### Sociodemographic Characteristics

Each site-specific common data file contained sociodemographic characteristics, including age, sex, race and ethnicity, socioeconomic status, and geography. Race and ethnicity (Hispanic ethnicity, non-Hispanic race including American Indian or Alaska Native, Asian or Pacific Islander, Black, multiracial, White) were determined at the time of an individual’s registration and are self-reported characteristics in most health systems.

### Statistical Analysis

We performed analyses using R Version 4.3.2 and Stata 15.1. Data were generated at each site using a standardized R script and aggregated at a central data coordinating site using R. To generate directly standardized prevalence rates and 95% confidence intervals, we used Stata’s *dstdize* function and adjusted for age categories (i.e., 18–24, 25–34, 35–44, 45–54, 55–64, 65–74, and 75 and older) and sex. We standardized estimates to the study sample, which is largely representative of the Minnesota adult population. Of note, recently incarcerated and homeless populations are nested populations within the general population and samples were not entirely independent though they were treated as such. Individuals younger than 18 or who were missing sex information (0.1%) were removed from the sample prior to analysis. For race and ethnicity estimates, we removed individuals with unknown or missing race and ethnicity information (8.1%).

## RESULTS

### Cohort Demographic Data

The cohort included 4,362,645 individuals, with the majority female (53%), White (73%). Our cohort was similar to the US Census-reported Minnesota adult population of approximately 4.5 million individuals. Among the total population, 0.5% (n = 20,139), 1.2% (n = 51,470), and 0.1% (n = 4,889) had recently experienced homelessness, jail incarceration, or prison incarceration, respectively. Individuals with recent homelessness were more likely to be younger (age 25–44, 48% vs. 34%), male (51% vs. 47%), and Black (34% vs. 8%) or American Indian/Alaska Native (11% vs. 1%) compared to the general population (Table 1). Similarly, individuals with recent jail or prison incarceration were more likely to be younger (age 25–44, 61% and 64%, respectively, vs. 34%), male (74% and 88%, respectively, vs. 47%), and Black (23% and 31%, respectively, vs. 8%) compared to the general population (Table 1).

**Table 1:** Sociodemographic Characteristics of Statewide Cohort—Minnesota, 2023

		**General Population**	**Recent Homelessness**	**Recent Jail Incarceration**	**Recent Prison Incarceration**
**N**		4,362,645	20,139	51,470	4,889
**Sex**					
	Male	47% (2,031,043)	51% (10,348)	74% (38,138)	88% (4,315)
	Female	53% (2,331,602)	49% (9,791)	26% (13,332)	12% (574)
**Age**					
	18–24	11% (482,500)	14% (2,883)	13% (6,566)	8% (395)
	25–34	17% (725,266)	23% (4,557)	31% (15,965)	30% (1,485)
	35–44	17% (743,904)	25% (5,063)	30% (15,365)	34% (1,663)
	45–54	14% (625,356)	17% (3,492)	15% (7,774)	16% (790)
	55–64	16% (693,514)	15% (3,104)	9% (4,405)	8% (412)
	65–74	14% (616,292)	4% (904)	2% (1,224)	2% (121)
	75 and older	11% (475,813)	1% (136)	0.3% (171)	0.5% (23)
**Race and Ethnicity**					
	American Indian/Alaska Native	1% (41,636)	11% (2,149)	7% (3,558)	8% (413)
	Asian or Pacific Islander	5% (205,403)	2% (384)	3% (1,492)	2% (104)
	Black	8% (348,564)	34% (6,757)	23% (11,944)	31% (1,516)
	Hispanic	5% (225,295)	5% (1,080)	7% (3,525)	7% (320)
	White	73% (3,186,964)	41% (8,180)	54% (27,717)	47% (2,279)
	Other/Missing/Unknown	8% (354,783)	8% (1,589)	6% (3,234)	5% (257)

### Physical Health, Mental Health, and Substance use Conditions Among People with Recent Homelessness

Adjusted for age and sex, prevalence of asthma (14.9% [95% CI, 14.1–15.6]) and chronic obstructive pulmonary disease (COPD) (10.5% [95% CI, 9.6–11.5]), were higher among individuals with recent homelessness, compared to the general population (Asthma: 7.1% [95% CI, 7.1–7.1] and COPD: 3.0% [95% CI, 3.0–3.0]). Rates of heart failure (6.6% [95% CI, 5.8–7.4] vs. 3.7% [95% CI, 3.6–3.7]), acute myocardial infarction (AMI) (4.9% [95% CI, 4.2, 5.6] vs. 2.1% [95% CI, 2.1–2.1]), and type II diabetes (14.9% [95% CI, 13.7-16.1] vs. 9.7% [95% CI, 9.7-9.8]) were also higher compared to the general population (Table 2).

**Table 2:** Prevalence of Physical Health, Mental Health, and Substance Use Conditions Among Statewide Cohort—Minnesota, 2023

	**General Population**		**Homeless**		**Recent Jail Incarceration**		**Recent Prison Incarceration**	
**Condition**	Prevalence (%)	95% CI	Prevalence (%)	95% CI	Prevalence (%)	95% CI	Prevalence (%)	95% CI
**Physical Health**								
Asthma	7.1%	7.1, 7.1	14.9%	14.1–15.6	9.6%	9.2, 10.1	10.1%	7.7, 12.5
COPD	3.0%	3.0, 3.0	10.5%	9.6, 11.5	6.1%	5.6, 6.6	5.5%	3.2, 7.8
Hypertension	33.4%	33.4, 33.5	37.3%	36.3, 38.4	37.8%	37.0, 38.7	26.3%	23.4 29.1
Hyperlipidemia	23.8%	23.7, 23.8	19.9%	18.7, 21.1	17.4%	16.7, 18.2	13.0%	10.5, 15.5
Diabetes mellitus, type 2	9.7%	9.7, 9.8	14.9%	13.7, 16.1	9.4%	8.8, 10.0	8.1%	6.2, 9.9
CAD/Ischemic heart disease	5.5%	5.5, 5.5	7.6%	6.8, 8.5	5.9%	5.3, 6.4	5.3%	4.3, 6.4
Heart failure	3.7%	3.6, 3.7	6.6%	5.8, 7.4	4.1%	3.6, 4.6	5.0%	2.8, 7.3
Stroke	2.5%	2.5, 2.5	4.4%	3.7, 5.1	2.8%	2.3, 3.2	2.6%	2.1, 3.2
AMI	2.1%	2.1, 2.1	4.9%	4.2, 5.6	3.2%	2.7, 3.7	3.2%	2.0, 4.4
Peripheral vascular disease	2.4%	2.4, 2.4	4.2%	3.4, 5.0	2.6%	2.1, 3.0	2.4%	0.3, 4.5
**Mental Health**								
Depression	17.2%	17.2, 17.3	37.4%	36.2, 38.6	30.6%	30.0, 31.3	24.7%	21.9, 27.4
Anxiety	22.5%	22.4, 22.5	43.7%	42.4, 44.9	37.3%	36.5, 38.1	30.2%	28.0, 32.3
PTSD	2.1%	2.1, 2.1	13.8%	13.1, 14.5	9.3%	8.9, 9.7	8.5%	7.2, 9.9
Bipolar disorder	1.6%	1.6, 1.6	11.8%	11.2, 12.3	8.1%	7.7, 8.5	8.0%	5.7, 10.2
Psychotic disorders	1.8%	1.7, 1.8	17.8%	17.0, 18.7	10.8%	10.3, 11.3	7.9%	6.8, 9.1
Suicide attempt/ideation	1.6%	1.6, 1.6	12.2%	11.6, 12.7	8.9%	8.5, 9.3	7.2%	6.0, 8.4
**Substance Use**								
Opioid	1.4%	1.4, 1.4	13.9%	13.3, 14.5	10.6%	10.2, 11.0	13.6%	11.2, 15.9
Alcohol	3.7%	3.7, 3.7	21.2%	20.4, 21.9	21.0%	20.4, 21.7	13.6%	11.1, 16.0
Hallucinogens	0.05%	0.05, 0.05	0.4%	0.3, 0.4	0.4%	0.3, 0.5	0.2%	0.1, 0.3
Methamphetamine	0.8%	0.8, 0.8	15.2%	14.5, 15.8	12.2%	11.9, 12.6	17.3%	14.8, 19.8
Cocaine	0.3%	0.3, 0.3	5.5%	5.1, 6.0	3.3%	3.0, 3.5	4.1%	2.0, 6.1
Cannabis	1.6%	1.5, 1.6	12.1%	11.6, 12.6	9.1%	8.8, 9.4	6.7%	5.5, 8.0

Estimates adjusted for age and sex

Abbreviation(s): COPD, chronic obstructive pulmonary disease. CAD, coronary artery disease. AMI, acute myocardial infarction. PTSD, post-traumatic stress disorder

We identified substantial differences in mental health and substance use condition prevalence between homeless and general populations. Depression (37.4% [95% CI, 36.2–38.6] vs. 17.2% [95% CI, 17.2–17.3]), anxiety (43.7% [95% CI, 42.4–44.9] vs. 22.5% [95% CI, 22.4–22.5]), post-traumatic stress disorder (PTSD) (13.8% [95% CI, 13.1–14.5] vs. 2.1% [95% CI, 2.1–2.1]), and psychotic disorders (17.8% [95% CI, 17.0–18.7] vs. 1.8% [95% CI, 1.7–1.8]) were higher among people with recent homelessness compared with the general population (Table 2). Similarly, opioid use disorder (13.9% [95% CI, 13.3–14.5] vs. 1.4% [95% CI, 1.4–1.4]), alcohol use disorder (21.2% [95% CI, 20.4–21.9] vs. 3.7% [95% CI, 3.7–3.7]), methamphetamine use disorder (15.2% [95% CI, 14.5–15.8] vs. 0.8% [95% CI, 0.8–0.8]), cocaine use disorder (5.5% [95% CI, 5.1–6.0] vs. 0.3% [95% CI, 0.3–0.3]), and cannabis use disorder (12.1% [95% CI, 11.6–12.6] vs. 1.6% [95% CI, 1.5–1.6]) were more common among people with recent homelessness compared with the general population (Table 2).

### Physical Health, Mental Health, and Substance use Conditions Among People with Recent Jail Or Prison Incarceration

Similar to health condition prevalence in individuals with recent homelessness, those with recent jail incarceration had a higher prevalence of several physical health, mental health and substance use conditions. Asthma (9.6% [95% CI, 9.2–10.1] vs. 7.1% [95% CI, 7.1–7.1]), depression (30.6% [95% CI, 30.0–31.3] vs. 17.2% [95% CI, 17.2–17.3]), anxiety (37.3% [95% CI, 36.5–38.1] vs. 22.5% [95% CI, 22.4–22.5]), psychotic disorders (10.8% [95% CI, 10.3–11.3] vs. 1.8% [95% CI, 1.7–1.8]), opioid use disorder (10.6% [95% CI, 10.2–11.0] vs. 1.4% [95% CI, 1.4–1.4]), alcohol use disorder (21.0% [95% CI, 20.4–21.7] vs. 3.7% [95% CI, 3.7–3.7]), methamphetamine use disorder (12.2% [95% CI, 11.9–12.6] vs. 0.8% [95% CI, 0.8–0.8]), cocaine use disorder (3.3% [95% CI, 3.0–3.5] vs. 0.3% [95% CI, 0.3–0.3]), and cannabis use disorder (9.1% [95% CI, 8.8–9.4] vs. 1.6% [95% CI, 1.5–1.6]) were higher among people with recent jail incarceration compared with the general population. Prevalence of peripheral vascular disease (2.6% vs. 2.4%), CAD/ischemic heart disease (5.9% vs. 5.4%), and stroke (2.8% vs. 2.3%) were similar among individuals with recent jail incarceration compared with the general population (Table 2).

Individuals with recent prison incarceration had higher rates of asthma (10.1% [95% CI, 7.7–12.5] and COPD (5.5% [95% CI, 3.2–7.8] compared to the general population (Asthma: 7.1% [95% CI, 7.1–7.1] and COPD: 3.0% [95% CI, 3.0–3.0]) and similar rates of other physical health conditions. Those with recent prison incarceration had higher prevalence of mental health and substance use conditions, including, depression (24.7% [95% CI, 21.9–27.4] vs. 17.2% [95% CI, 17.2–17.3]), anxiety (30.2% [95% CI, 28.0–32.3] vs. 22.5% [95% CI, 22.4–22.5]), bipolar disorder (8.0% [95% CI, 5.7–10.2] vs. 1.6% [95% CI, 1.6–1.6]), opioid use disorder (13.6% [95% CI, 11.2–15.0] vs. 1.4% [95% CI, 1.4–1.4]), and methamphetamine use disorder (17.3% [95% CI, 14.8–19.8] vs. 0.8% [95% CI, 0.8–0.8]), compared to the general population (Table 2).

### Racial and Ethnic Disparities Among People with Recent Homelessness

We identified important racial differences in health condition prevalence among individuals with recent homelessness. Black individuals with recent homelessness had the highest proportion of asthma (18.0% [95% CI, 16.0–20.0]) and hypertension (36.8% [95% CI, 34.5-39.1]) among people with recent homelessness. Type 2 diabetes was higher among Minnesotans with recent homelessness who identified as Hispanic (17.8% [95% CI, 14.4-21.2]), Asian/Pacific Islander (17.0% [95% CI, 11.8-22.1), Black (14.7% [95% CI, 12.7-16.7]), or Native American/Alaska Native (15.0% [95% CI, 12.7-17.2]) compared to White individuals (11.6% [95% CI, 10.6-12.6]) with recent homelessness (Table 3).

**Table 3:** Prevalence of Health Conditions Among Individuals with Recent Homelessness by Race and Ethnicity—Minnesota, 2023

**Condition**	**White**		**Black**		**Native American/Alaska Native**		**Hispanic**			**Asian/Pacific Islander**	
	Prevalence (%)	95% CI	Prevalence (%)	95% CI	Prevalence (%)	95% CI	Prevalence (%)	95% CI		Prevalence (%)	95% CI
**Physical Health**											
Asthma	14.4%	13.4, 15.4	18.0%	16.0, 20.0	10.4%	8.8, 11.9	13.9%	8.6, 19.3	10.1%		6.6, 13.6
COPD	11.7%	10.7, 12.8	7.0%	5.5, 8.5	6.4%	4.7, 8.1	6.5%	1.3, 11.7	7.0%		2.1, 11.8
Hypertension	35.4%	34.2, 36.5	36.8%	34.5, 39.1	24.5%	22.0, 27.0	33.8%	31.4, 36.3	28.8%		23.4, 34.1
Hyperlipidemia	18.7%	17.5, 19.9	17.8%	15.6, 20.0	11.6%	9.6, 13.5	15.4%	9.9, 20.9	18.5%		13.0, 24.0
Diabetes mellitus, type 2	11.6%	10.6, 12.6	14.7%	12.7, 16.7	15.0%	12.7, 17.2	17.8%	14.4, 21.2	17.0%		11.8, 22.1
CAD/Ischemic heart disease	7.1%	6.2, 8.0	7.7%	6.1, 9.4	6.0%	4.4, 7.6	3.8%	2.4, 5.3	6.4%		3.2, 9.5
Heart failure	6.0%	5.2, 6.8	6.7%	5.4, 8.1	3.9%	2.7, 5.1	2.7%	1.4, 3.9	5.7%		2.6, 8.7
Stroke	4.1%	3.3, 4.8	4.5%	3.1, 5.9	4.5%	3.0, 5.9	3.0%	1.7, 4.3	4.0%		1.2, 6.9
AMI	4.8%	4.0, 5.5	5.5%	4.2, 6.8	3.9%	2.6, 5.1	3.4%	2.0, 4.8	3.7%		0.9, 6.5
Peripheral vascular disease	3.3%	2.6, 3.9	2.6%	1.7, 3.5	2.6%	1.5, 3.7	1.5%	0.6, 2.5	1.6%		0, 3.7
**Mental Health**											
Depression	40.5%	39.2, 41.8	31.9%	29.9, 34.0	26.6%	24.4, 28.9	32.4%	26.7, 38.1	33.7%		28.0, 39.5
Anxiety	48.7%	47.4, 50.1	33.2%	31.3, 35.1	33.3%	31.0, 35.5	40.5%	34.7, 46.2	34.8%		28.7, 40.8
PTSD	14.9%	14.1, 15.7	11.4%	10.4, 12.3	11.2%	9.9, 12.5	12.0%	10.2, 13.8	11.1%		7.7, 14.5
Bipolar disorder	14.2%	13.3, 15.0	10.0%	9.0, 11.0	6.0%	4.9, 7.0	9.7%	8.0, 11.4	6.3%		4.3, 8.3
Psychotic disorders	16.2%	15.2, 17.1	18.7%	17.9, 20.5	15.0%	13.5, 16.6	14.3%	12.2, 16.4	15.5%		11.9, 19.2
Suicide attempt/ideation	14.2%	13.3, 15.0	9.3%	8.4, 10.1	9.6%	8.2, 11.0	10.4%	8.7, 12.1	9.2%		6.6, 11.9
**Substance Use**											
Opioid	13.4%	12.7, 14.2	11.2%	9.7, 12.7	20.3%	18.4, 22.1	10.5%	8.7, 12.2	16.4%		12.3, 20.5
Alcohol	21.7%	20.6, 22.8	18.7%	17.4, 20.1	24.2%	22.2, 26.3	17.1%	14.9, 19.3	13.0%		9.1, 16.8
Hallucinogens	0.3%	0.2, 0.4	0.7%	0.5, 0.8	0.1%	0, 0.2	0.2%	0, 0.4	0.0%		0, 0
Methamphetamine	18.6%	17.7, 19.4	6.9%	6.3, 7.6	18.4%	16.7, 20.0	13.6%	11.7, 15.5	16.4%		12.8, 20.0
Cocaine	3.4%	2.9, 3.9	10.5%	9.0, 12.1	3.1%	2.3, 3.9	3.3%	2.1, 4.5	2.1%		0.9, 3.4
Cannabis	12.3%	11.6, 13.1	12.7%	11.7, 13.7	10.0%	8.5, 11.5	9.5%	7.9, 11.2	6.1%		4.0, 8.3

Estimates adjusted for age and sex

Abbreviation(s): COPD, chronic obstructive pulmonary disease. CAD, coronary artery disease. AMI, acute myocardial infarction. PTSD, post-traumatic stress disorder

White Minnesotans with recent homelessness had a greater proportion of depression (40.5% [95% CI, 39.2–41.8]) and anxiety (48.7% [95% CI, 47.4–50.1]) compared to Black (31.9% [95% CI, 29.9–34.0]; 33.2% [95% CI, 31.3–35.1], respectively), Native American/Alaska Native (26.6% [95% CI, 24.4–28.9] and 33.3% [95% CI 31.0–35.5], respectively), and Hispanic individuals with recent homelessness (32.4% [95% CI, 26.7–38.1]; 40.5% [95% CI, 34.7–46.2], respectively). Black individuals with recent homelessness had the highest prevalence of psychotic disorders (18.7% [95% CI, 17.9–20.5]) compared with White (16.2% [95% CI, 15.2–17.1]), Native American/Alaska Native (15.0% [95% CI, 13.5–16.6), Hispanic (14.3% [95% CI, 12.2–16.4]), and Asian/Pacific Islander individuals with recent homelessness (9.2% [95% CI, 6.6–11.9).

Native American/Alaska Native individuals with recent homelessness had higher rates of opioid use (20.3% [95% CI, 18.4–22.1]) compared to White (13.4%, [95% CI, 12.7–14.2]), Black (11.2% [95% CI, 9.7–12.7]) and Hispanic (10.5% [95% CI, 8.7–12.2]) individuals with recent homelessness (Table 3).

### Racial and Ethnic Disparities Among People with Recent Jail or Prison Incarceration

Black individuals with recent jail incarceration had higher rates of asthma (12.7% [95% CI, 11.3–14.2]) compared to White (9.1% [95% CI, 8.5–9.6]), Native American/Alaska Native (8.1% [95% CI, 6.2–10.0]), Hispanic (7.9% [95% CI, 6.3–9.4]), and Asian/Pacific Islander (7.9% [95% CI, 2.6–13.1]) individuals with recent incarceration. Hypertension was highest among White and Hispanic individuals with recent jail incarceration (36.2% [95% CI, 35.3-37.0] and 37.6% [95% CI, 35.0-40.1], respectively) compared to Native Americans (28.9% [95% CI, 25.0-32.0) or Asian/Pacific Islanders (31.9% [95% CI, 28.7-35.0]) (Table 4).

**Table 4:** Prevalence of Health Conditions among Individuals with Recent Jail Incarceration by Race and Ethnicity—Minnesota, 2023

**Condition**	**White**		**Black**		**Native American/Alaska Native**			**Hispanic**		**Asian/Pacific Islander**	
	Prevalence (%)	95% CI	Prevalence (%)	95% CI	Prevalence (%)	95% CI		Prevalence (%)	95% CI	Prevalence (%)	95% CI
**Physical Health**											
Asthma	9.1%	8.5, 9.6	12.7%	11.3, 14.2	8.1%		6.2, 10.0	7.9%	6.3, 9.4	7.9%	2.6, 13.1
COPD	6.1%	5.5, 6.7	6.0%	4.2, 7.7	4.3%		2.4, 6.2	2.8%	1.4, 4.3	0.3%	0, 0.6
Hypertension	36.2%	35.3, 37.0	33.7%	31.8, 35.5	28.9%		25.0, 32.9	37.6%	35.0, 40.1	31.9%	28.7, 35.0
Hyperlipidemia	17.0%	16.3, 17.8	15.0%	12.9, 17.1	10.8%		7.5, 14.1	15.5%	10.0, 21.0	14.4%	8.2, 20.6
Diabetes mellitus, type 2	7.9%	7.3, 8.6	11.3%	9.8, 12.8	14.8%		11.2, 18.4	12.5%	10.2, 14.8	10.6%	7.9, 13.3
CAD/Ischemic heart disease	5.1%	4.6, 5.7	5.5%	3.7, 7.2	4.4%		2.2, 6.6	3.2%	1.8, 4.6	1.7%	0.2, 3.1
Heart failure	3.8%	3.4, 4.3	3.9%	2.8, 5.0	3.3%		2.1, 4.4	1.8%	1.0, 2.6	2.4%	0.9, 4.0
Stroke	2.3%	2.0, 2.7	2.5%	1.6, 3.4	3.0%		2.0, 4.1	1.3%	0.6, 2.0	2.0%	0.5, 3.5
AMI	2.6%	2.2, 3.0	4.1%	2.7, 5.6	2.9%		1.8, 4.0	1.6%	0.7, 2.4	1.5%	0.04, 2.9
Peripheral vascular disease	2.3%	1.9, 2.7	1.6%	0.8, 2.5	3.6%		1.1, 6.1	1.5%	0.5, 2.5	0.2%	0, 0.5
**Mental Health**											
Depression	32.9%	32.0, 33.7	24.8%	22.8, 26.7	21.6%		19.4, 23.9	23.2%	20.9, 25.4	13.0%	10.8, 15.3
Anxiety	40.2%	39.4, 41.1	28.7%	26.5, 30.8	34.7%		31.8, 37.6	29.0%	26.6, 31.4	19.0%	13.5, 24.5
PTSD	9.5%	9.0, 10.0	9.4%	7.9, 10.9	9.2%		7.4, 11.0	7.3%	5.9, 8.8	4.0%	2.9, 5.1
Bipolar disorder	8.6%	8.1, 9.0	8.2%	6.8, 9.6	4.7%		3.8, 5.6	5.6%	4.3, 6.9	7.1%	2.0, 12.2
Psychotic disorders	9.4%	8.9, 9.9	14.2%	12.6, 15.9	13.1%		10.7, 15.5	8.5%	6.9, 10.1	6.7%	4.8, 8.5
Suicide attempt/ideation	9.6%	9.1, 10.1	7.4%	6.2, 8.6	9.7%		7.8, 11.6	8.0%	6.5, 9.4	6.9%	5.0, 8.9
**Substance Use**											
Opioid	10.0%	9.6, 10.5	10.7%	9.4, 12.0	17.2%		14.9, 19.5	7.8%	6.4, 9.2	4.8%	3.7, 5.9
Alcohol	22.0%	21.2, 22.7	18.1%	16.3, 20.0	22.5%		19.1, 25.9	18.1%	12.7, 23.5	12.1%	9.0, 15.3
Hallucinogens	0.5%	0.3, 0.6	0.6%	0.5, 0.8	0.1%		0.02, 0.1	0.2%	0.1, 0.4	0.1%	0, 0.2
Methamphetamine	13.6%	13.1, 14.1	6.2%	5.4, 7.1	18.4%		16.3, 20.4	10.9%	9.4, 12.5	8.0%	6.7, 9.3
Cocaine	2.3%	2.0, 2.5	9.4%	7.9, 10.9	2.2%		1.6, 2.9	2.8%	1.8, 3.8	1.4%	0.7, 2.0
Cannabis	9.1%	8.6, 0.5	10.6%	9.8, 11.5	9.0%		7.1, 11.0	6.2%	5.3, 7.1	4.3%	3.2, 5.4

Estimates adjusted for age and sex

Abbreviation(s): COPD, chronic obstructive pulmonary disease. CAD, coronary artery disease. AMI, acute myocardial infarction. PTSD, post-traumatic stress disorder

White individuals with recent jail incarceration had higher rates of depression (32.9% [95% CI, 32.0–33.7]) and anxiety (40.2% [95% CI, 39.4–41.1]) when compared to other racial and ethnic groups. Black individuals with recent jail incarceration had the highest prevalence of psychotic disorders (14.2% [95% CI 12.6–15.9]) compared to other racial and ethnic groups with recent jail incarceration. Native American/Alaska Native individuals with recent jail incarceration had the highest prevalence of opioid (17.2% [95% CI, 14.9–19.5]) and methamphetamine use disorder (18.4% [95% CI, 16.3–20.4]) compared to all other racial and ethnic groups with recent incarceration (Table 4).

White individuals with recent prison incarceration had higher prevalence of COPD (7.1%, [95% CI, 3.7–10.6]) compared to Black (1.5% [95% CI, 0.6–2.5]), Native American/Alaska Native (1.0% [0–2.5]), and Hispanic (0.4% [95% CI, 0–1.1]) individuals with recent prison incarceration. Black individuals with recent prison incarceration had highest rates of psychotic disorders (12.7% [95% CI, 8.6–16.9]) compared to individuals of all other races with recent prison incarceration. Finally, Black individuals with recent incarceration (15.6%, [95% CI, 11.5–19.8]) had higher rates of cocaine use disorder compared with White (6.2% [95% CI, 4.5–7.9]), Native American/Alaska Native (6.6% [95% CI, 2.3–10.9]), Hispanic (9.1% [95% CI, 5.1–13.1]), and Asian/Pacific Islander (4.5% [95% CI, 0.8–8.2]) individuals with recent prison incarceration (Table 5).

**Table 5:** Prevalence of Health Conditions among Individuals with Recent Prison Incarceration by Race and Ethnicity—Minnesota, 2023

**Condition**	**White**		**Black**		**Native American/Alaska Native**		**Hispanic**		**Asian/Pacific Islander**	
	Prevalence (%)	95% CI	Prevalence (%)	95% CI	Prevalence (%)	95% CI	Prevalence (%)	95% CI	Prevalence (%)	95% CI
**Physical Health**										
Asthma	10.2%	7.9, 12.6	16.6%	12.9, 20.2	5.3%	2.4, 8.2	11.1%	4.4, 17.9	2.8%	0.0, 6.3
COPD	7.1%	3.7, 10.6	1.5%	0.6, 2.5	1.0%	0, 2.5	0.4%	0, 1.1	0.0%	0, 0
Hypertension	26.8%	22.7, 31.0	26.8%	19.6, 34.0	18.1%	12.3, 24.0	21.4%	14.7, 28.1	14.5%	8.8, 20.1
Hyperlipidemia	14.0%	10.2, 17.8	13.2%	9.2, 17.2	8.4%	3.5, 13.2	3.0%	1.4, 4.5	7.9%	2.3, 13.5
Diabetes mellitus, type 2	7.7%	4.8, 10.7	10.5%	6.2, 14.8	9.6%	4.6, 14.6	2.7%	1.4, 4.1	4.7%	1.5, 8.1
CAD/Ischemic heart disease	4.5%	3.2, 5.8	3.0%	1.2, 4.9	3.0%	0.4, 5.7	1.2%	0.03, 2.4	3.8%	0, 8.5
Heart failure	5.1%	1.8, 8.4	4.8%	1.7, 7.9	2.7%	0.8, 4.6	0.5%	0, 1.2	0.0%	0, 0
Stroke	1.4%	0.7, 2.1	1.6%	0.6, 2.6	1.4%	0.1, 2.8	0.6%	0, 1.4	0.9%	0, 1.9
AMI	2.6%	1.6, 3.7	2.0%	0.7, 3.3	2.1%	0, 4.1	1.4%	0, 2.9	1.3%	0.02, 2.6
Peripheral vascular disease	1.0%	0.4, 1.5	0.6%	0.03, 1.2	1.0%	0, 2.2	0.4%	0, 1.1	0.0%	0, 0
**Mental Health**										
Depression	28.8%	24.7, 32.8	22.7%	17.0, 28.4	20.4%	15.0, 25.9	24.6%	17.1, 32.1	9.2%	4.6, 13.9
Anxiety	32.5%	30.7, 36.4	25.5%	19.7, 31.3	27.4%	21.7, 33.1	33.6%	26.0, 41.1	19.8%	14.6, 25.1
PTSD	8.9%	7.0, 10.9	9.3%	4.9, 13.8	6.7%	4.3, 9.1	11.0%	5.7, 16.4	2.0%	0.4, 3.7
Bipolar disorder	8.2%	6.2, 10.2	12.1%	9.8, 14.4	2.7%	1.0, 4.3	10.3%	4.9, 15.8	9.0%	9.0, 9.0
Psychotic disorders	6.5%	5.3, 7.8	12.7%	8.6, 16.9	5.2%	3.2, 7.2	7.0%	3.0, 11.1	7.0%	2.2, 11.7
Suicide attempt/ideation	6.9%	5.4, 8.4	6.8%	3.1, 10.5	11.3%	6.6, 16.0	12.3%	5.5, 19.1	11.6%	9.8, 13.4
**Substance Use**										
Opioid	13.3%	11.2, 15.4	17.1%	14.3, 19.9	16.1%	11.0, 21.2	10.8%	6.6, 14.9	7.2%	2.4, 11.9
Alcohol	12.0%	10.2, 13.9	22.6%	17.4, 27.8	18.5%	12.9, 24.1	11.2%	5.1, 17.3	11.2%	9.5, 12.9
Hallucinogens	0.1%	0.02, 0.3	0.3%	0.2, 0.5	0.0%	0, 0	0.2%	0, 0.5	0.0%	0, 0
Methamphetamine	20.4%	16.5, 24.3	8.8%	4.4, 13.2	21.5%	16.2, 26.7	17.6%	12.0, 23.3	13.2%	7.4, 18.9
Cocaine	2.6%	2.3, 3.9	15.6%	11.5, 19.8	1.1%	0, 2.3	3.5%	0, 7.1	0.3%	0, 0.7
Cannabis	6.2%	4.5, 7.9	9.9%	5.7, 14.1	6.6%	2.3, 10.9	9.1%	5.1, 13.1	4.5%	0.8, 8.2

Estimates adjusted for age and sex

Abbreviation(s): COPD, chronic obstructive pulmonary disease. CAD, coronary artery disease. AMI, acute myocardial infarction. PTSD, post-traumatic stress disorder

## DISCUSSION

Through linkage of statewide EHR and administrative data, we produced timely estimates of physical health, mental health, and substance use conditions among Minnesotans with recent homelessness or incarceration. Our findings highlight the substantial severe and multiple disadvantages faced by these populations; policy reforms are needed to ensure that necessary medical and social support can be accessed by these populations and that such care is financially sustainable. Our findings were produced through a linkage process that is scalable and could be replicated in additional jurisdictions to produce actionable data that drives care for people with severe and multiple disadvantages.

The prevalence of health conditions by race and ethnicity in individuals with recent homelessness or incarceration varied in important ways. For example, the prevalence of mental depression and anxiety were higher among White individuals with recent homelessness or jail incarceration compared with other racial and ethnic groups, while psychotic disorders were higher among Black individuals with recent homelessness or incarceration. Our estimates of physical health conditions among individuals with recent incarceration, such as COPD, hypertension, diabetes, and ischemic heart disease, were higher than similar analyses of claims from Hennepin County but identified similar racial disparities and trends.^18^ Finally, we found notably high rates of psychotic disorders, opioid use disorder, and methamphetamine use disorder among Native American Minnesotans, which has been previously reported.^19,20^

We found EHR-based depression prevalence among individuals with recent jail incarceration was higher compared with self-reported national estimates of people with recent criminal justice involvement (30.6% vs. 13.9%)^21^ but lower compared with estimates using linked healthcare claims data among people on probation in Hennepin County, Minnesota (30.6% vs. 41.6%).^18^ Compared with EHR-based estimates of health conditions among individuals with a history of homelessness in Ohio,^6^ we found lower rates of hypertension (37.3% vs 44.2%), diabetes (14.9% vs. 19.2%), heart failure (6.6% vs. 8.4%), depression (37.4% vs. 48.1%), and alcohol use disorder (21.2% vs. 30.6%). Finally, our estimates of mental health and substance use conditions among people with recent homelessness or incarceration were higher compared with recent systematic reviews assessing behavioral prevalence in these populations.^7,22,23^ We hypothesize the higher prevalence we identified may be related to improved diagnosis rates in the community compared with individuals who are receiving care during incarceration or because our population, by definition, is a population that is using healthcare and may be sicker at baseline.

Our report offers a path forward for generating prevalence estimates among populations with severe and multiple disadvantages. Because health condition data among people with recent homelessness and incarceration have historically been challenging to generate, there is often minimal information to guide healthcare policy and delivery for these populations. As more states expand their Medicaid programs to cover housing resources and medical services during incarceration, data systems that can be used for program evaluation are critical. For example, dozens of states are in the process of expanding Medicaid to people in prisons and jails through Medicaid 1115 Reentry Waivers.^24–26^ The processes and estimates we outline here could be scaled to inform the CMS-mandated evaluations of these programs. Importantly, the underlying data infrastructure for this work is built on an open-source, international common data model using linkage processes that can be used in a variety of contexts.^27^

Our work has important limitations. This cohort only includes Minnesotans who had a medical encounter within one of the participating health systems during a 3-year period, although this represents over 95% of the Minnesota adult population based on 2022 US Census estimates. The specific health condition estimates may not generalize to other states, though the general trends reported here are likely to be similar in different jurisdictions. Second, we capture health conditions that have been diagnosed in a medical record. This leads to conservative estimates of health conditions, particularly substance use disorders, given variable accuracy in ICD-10 coding.^28–30^ Future work among our group aims to deploy natural language processing to capture additional information from the free text of clinical documentation. Third, we use a privacy preserving record linkage to deduplicate and link external data sources to EHR data. Through this process we may slightly underreport homelessness and incarceration; our best estimate is around 5–10% underreported. This would make our prevalence estimates somewhat more conservative. Finally, our measures of incarceration and homelessness include any within the past year and do not differentiate between different levels of service use within HMIS.

## CONCLUSION

In this report, we utilized linked EHR and administrative data to provide statewide estimates of physical health, mental health, and substance use conditions for Minnesotans with recent homelessness or criminal justice involvement. To our knowledge, these data represent the first EHR-based statewide health condition estimates among these populations. We highlight important disparities between people with recent homelessness and incarceration compared with the general population, particularly with regards to mental health and substance use conditions. The MNEHRC data model represents an efficient, collaborative approach to providing real-time public health data, including information on people with recent homelessness or incarceration.

## Data Availability

All pertinent data and materials are available upon reasonable request.
